# Supplementary material for: Dichloroacetate Radiosensitizes Hypoxic Breast Cancer Cells
Source: Int J Mol Sci. 2020 Dec 9;21(24):9367. doi: 10.3390/ijms21249367 (PMC7763818; doi:10.3390/ijms21249367)
Supplement: Supplementary file 1 [file ijms-21-09367-s001.pdf]

Supplementary Information

Dichloroacetate radiosensitizes hypoxic breast cancer cells

de Mey et al.

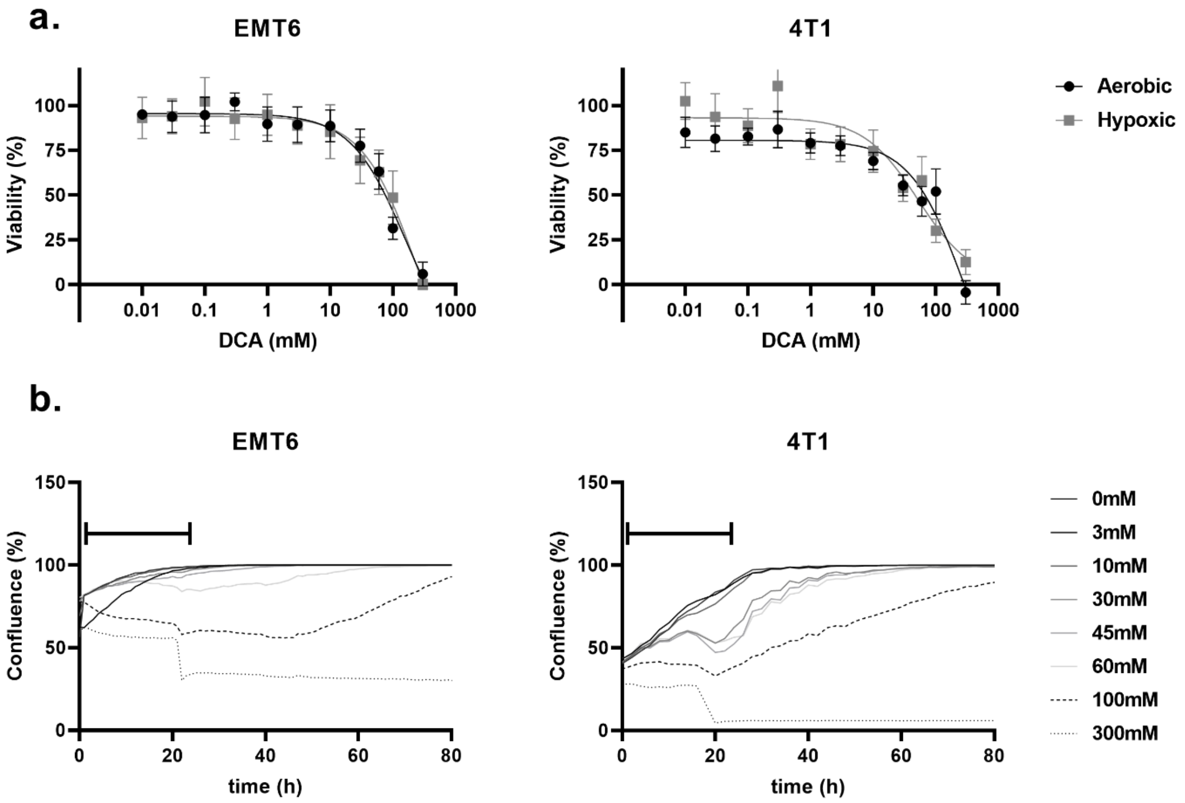

Figure S1: Growth of EMT6 and 4T1 breast cancer cells after treatment with DCA at indicated conditions. (**a**) Cell viability was assessed by MTT assay after treatment with DCA, and IC_{50} was calculated accordingly. (**b**) Cell proliferation was evaluated by live-confluence measurement with the Incucyte system. Treatment time of DCA is indicated with the capped line.

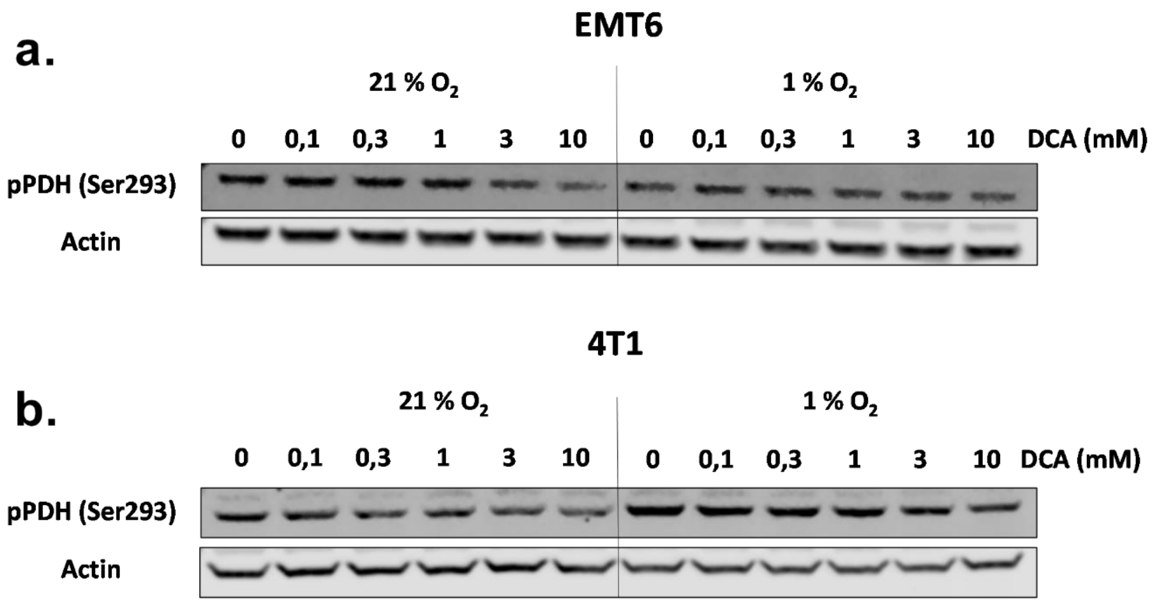

Figure S2: DCA decreases phosphorylated PDH of TNBC cells. Representative western blot of p-PDH (Ser293) in EMT6 (a) and 4T1 (b) cells, after treatment with DCA (0.1mM, 0.3mM, 1mM, 3mM and 10mM) under aerobic and hypoxic conditions.

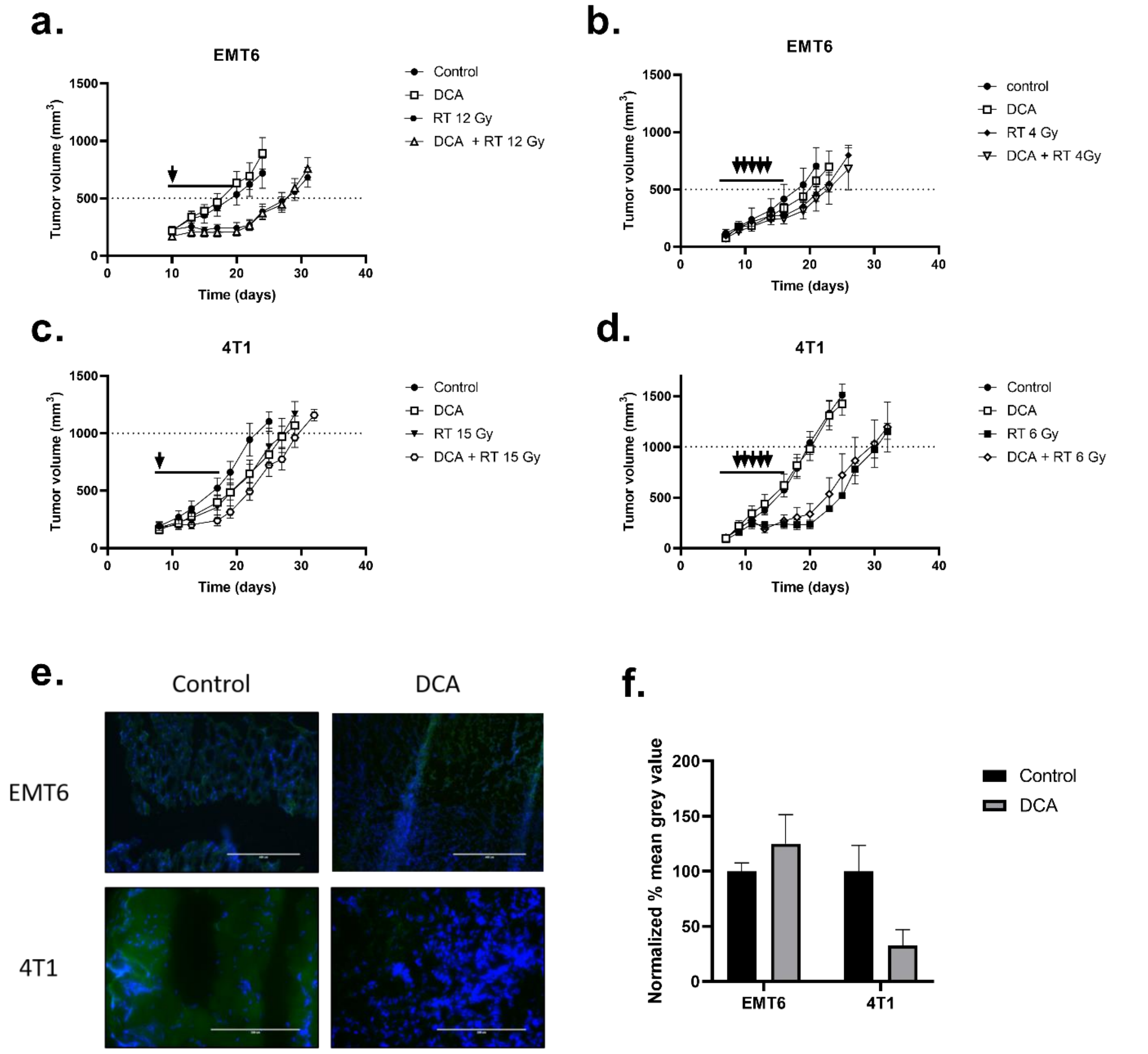

Figure S3: DCA fails to radiosensitize tumour cells *in vivo*. Tumour growth of EMT6 tumours in Balb/c mice administered with DCA 300mg/kg (ip. or it.) for 10 consecutive days and single dose radiation (12Gy) (**a**) or fractionated radiation (5*4Gy) (**b**). Tumour growth of 4T1 tumours in Balb/c mice daily for treated with DCA 300mg/kg (ip.) for 10 consecutive days and single fraction radiation of 15Gy (**c**), or treated with fractionated radiation of 5*6Gy (**d**). (**e**) Representative pictures and quantified data (**f**) of pimonidazole and dapi staining of EMT6 and 4T1 tumours.

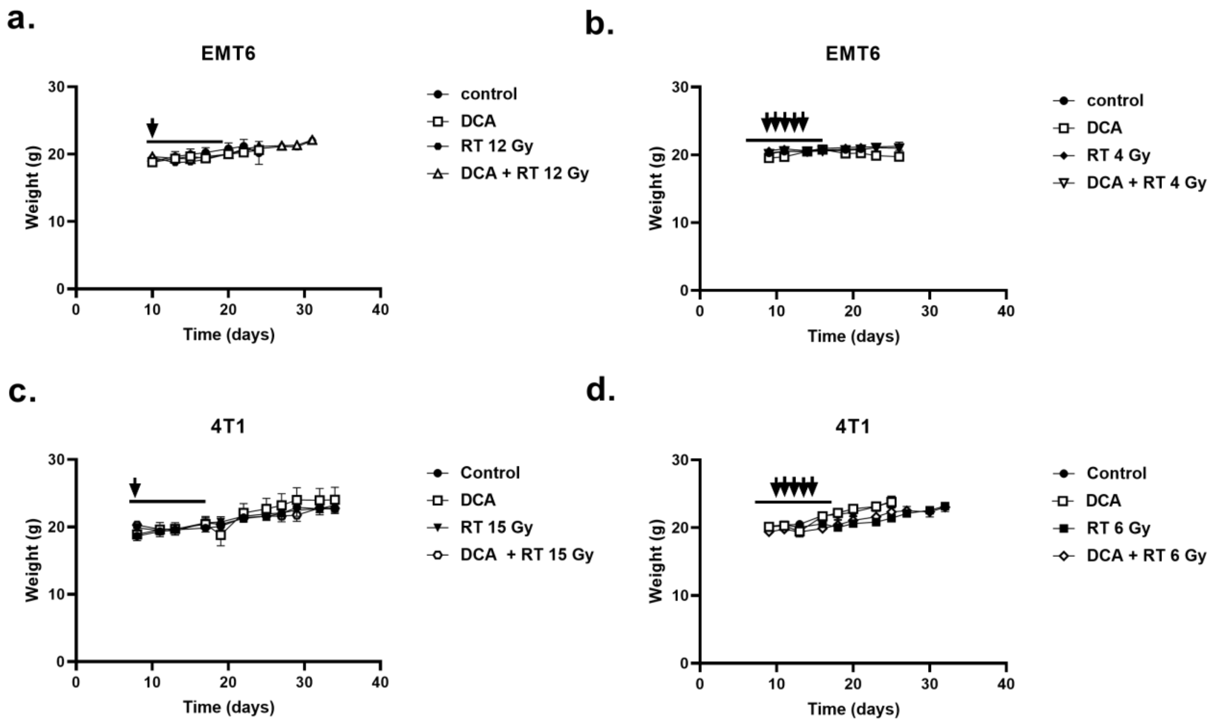

Figure S4: DCA and radiotherapy treatment does not cause toxicity in the mice. Weight of EMT6 inoculated Balb/c mice mice treated with DCA 300mg/kg (i.p. or i.t.) for 10 consecutive days and single dose radiation (12Gy) (a). Weight of EMT6 inoculated Balb/c mice mice treated with DCA 300mg/kg (i.p. or i.t.) for 10 consecutive days and fractionated radiation (5*4Gy) (b). Weight of 4T1 inoculated Balb/c mice treated with DCA 300mg/kg (i.p.) for 10 consecutive days and single fraction radiation of 15Gy (c). Weight of 4T1 inoculated Balb/c mice treated with DCA 300mg/kg (i.p.) for 10 consecutive days and treated with fractionated radiation of 5*6Gy (d).
